# Collective Catering Activities and Official Controls: Dietary Promotion, Sustainability and Future Perspectives

**DOI:** 10.3390/healthcare11091347

**Published:** 2023-05-07

**Authors:** Vincenzo Marcotrigiano, Giacomo Domenico Stingi, Prudenza Tiziana Nugnes, Sabrina Mancano, Vita Maria Lagreca, Teresa Tarricone, Gerardo Salerno, Pietro Pasquale, Paola Marchet, Giovanni Andrea Sava, Alessandro Citiulo, Monica Tissi, Stefania Oliva, Sandro Cinquetti, Christian Napoli

**Affiliations:** 1 Prevention Department, Local Health Authority BT, Barletta-Andria-Trani, 76125 Trani, Italy; giacomodomenico.stingi@aslbat.it; 2 Food Hygiene and Nutrition Service, Prevention Department, Local Health Authority BT, Barletta-Andria-Trani, 76125 Trani, Italy; tiziana.nugnes@aslbat.it (P.T.N.); sabrina.mancano@aslbat.it (S.M.); vitamaria.lagreca@aslbat.it (V.M.L.); teresa.tarricone@aslbat.it (T.T.); 3 Department of Neurosciences, Mental Health and Sensory Organs “NESMOS”, Sapienza University of Rome, 00189 Rome, Italy; gerardo.salerno@uniroma1.it; 4 Apulia Region, Health and Wellness Promotion Section, Food Hygiene and Preventive Nutrition Organizational Structure, 70126 Bari, Italy; p.pasquale@regione.puglia.it; 5 Prevention Department, Local Health Authority “AULSS 1 Dolomiti”, 32100 Belluno, Italy; paola.marchet@aulss1.veneto.it (P.M.); sandro.cinquetti@aulss1.veneto.it (S.C.); 6 Food Hygiene and Nutrition Service, Prevention Department, Local Health Authority “AULSS 1 Dolomiti”, 32100 Belluno, Italy; giovanni.sava@aulss1.veneto.it (G.A.S.); alessandro.citiulo@aulss1.veneto.it (A.C.); monica.tissi@aulss1.veneto.it (M.T.); 7 Department of Public Health and Infectious Diseases, Sapienza University of Rome, Piazzale Aldo Moro 5, 00185 Rome, Italy; stefania.oliva@uniroma1.it; 8 Department of Medical Surgical Sciences and Translational Medicine, Sapienza University of Rome, 00189 Rome, Italy; christian.napoli@uniroma1.it

**Keywords:** public health, official control, collective catering, nutrition, sustainability

## Abstract

Ensuring safe meals with suitable hygienic-sanitary and nutritional features is an essential requirement to guarantee health in different settings. This study aims to evaluate the compliance of collective catering menus adopted in both school canteens and healthcare facilities in a regional area where specific guidelines have been issued, assessing many matters from food weight to single courses and from the use of wholegrain pasta and bread to the rotation of seasonal fruit and vegetables. Overall, 85 menus, edited by freelance professionals and endorsed by the Food Hygiene and Nutrition Service staff of the Local Health Authority, were assessed from 2018 to 2022, highlighting critical issues potentially attributable at a local level to the lack of complete knowledge of the existence of guidelines and official reference documents among nutrition professionals. Since the preliminary outcomes show non-compliance in both sectors investigated, it is essential to continue to strengthen the role of prevention departments entrusted with services dedicated to food and nutritional safety and promote joint official controls performed by healthcare workers and other professionals with different backgrounds in order to ensure safe food for the target population that use collective catering services. In school canteens and healthcare facilities, providing and administering food is an opportunity to promote health through a balanced diet and safe food and offers opportunities for the development of community well-being and the local economy in a sustainable manner, understood in economic, environmental and social terms.

## 1. Introduction

In recent years, owing to an increase in the prevalence of obesity in the developmental age population, with a consequential growing risk of long-term complications, the adoption of timely preventive actions is required to support the availability of healthy foods and balanced nutrition throughout the life cycle [1,2]. The “life-course” approach, promoted by the World Health Organization, is expressly referred to in the current Italian National Prevention Plan 2020–2025, outlining the implementation of health promotion programs along with regional prevention plans [3,4]. Surveillance systems such as “OKkio alla salute”, “HBSC” and “Passi” provide documentary evidence that unhealthy lifestyles are widespread in the Italian population in all age groups [5,6].

Since many behaviors, which will become permanent in adults, begin to take shape at the developmental age, it is necessary to define effective interventions, programs and policies for health education and promotion, especially in this population group. As widely reported in the literature, a combination of approaches implemented by a multiplicity of actors is essential in order to successfully fight the high burden of non-communicable diseases (NCDs). Their effectiveness will be strengthened by actions on equity and health determinants and the empowerment of the target population [7].

Recent evidence indicates that the best results of health promotion actions on the target developmental age population are obtained at school. These contexts certainly have a priority role in educational terms, not only for aspects related to culture and education, but for the promotion of healthy and correct lifestyles too [8,9]. Indeed, school catering has been identified as a priority tool for promoting health and educating on correct nutrition, involving children, families and teachers simultaneously [10,11].

Likewise, collective catering carried out in healthcare facilities, including hospitals and care homes, must address the needs of specific population targets, which are often heterogeneous, by considering comorbidities and the need to provide special diets. Therefore, health authorities must ensure a suitable control system for both nutritional and hygienic sanitary features of the whole food chain, from production to transport and consumption.

In addition, the potential adherence to voluntary certification schemes, such as the UNI EN ISO IEC 9001:2015, UNI EN ISO IEC 22000:2018 and UNI EN ISO IEC 22005:2008 standards, could be useful for raising the safety and quality requirements of food safety management systems [12,13,14]. These standards specifically refer to the requirements of quality management systems, food chain organizations and traceability design and implementation. The UNI Agro-food Sector and Food Service Outside The Home Technical Commissions compiled the UNI 11584:2021 standard on the minimum requirements for the design of menus intended for collective, public and private catering by public bodies, companies and professionals [15]. The standard aim focuses on the process relating to the planning of menus and to the use of objective, measurable and generally valid elements within the framework of defined principles affecting food safety and health. It also considers communities and individual needs, focusing on the principles inspired by sustainable development, energy saving and waste food reduction, as well as the protection of consumer interests. In its complexity, menu drafting requires technical planning that takes into account scientific and economic features; hence, its elaboration by a multidisciplinary team, dedicated to sharing knowledge and the needs of all stakeholders, is essential. Moreover, the close correlation between human, animal and environmental health in the One Health approach is widely agreed upon; environment protection can be promoted with the aid of sustainable diets, a respect for seasonality, the rotation of foods offered and a reduction in food waste, with a consequent improvement in living conditions for individuals who consume such foods.

In this context, new regional guidelines for schools, companies and hospital caterers have been issued in the Puglia region in Southern Italy [16,17]. The main purpose of the guidelines is to facilitate and support the adoption from childhood of correct eating habits for health promotion and for the prevention of NCDs—for which incorrect nutrition is one of the main risk factors—and to meet the need for parallel useful recommendations to ensure food safety.

According to the guidelines, in the Italian tradition, lunch is generally made up of a first course, a second course, a side dish and fruit. This distribution can be maintained in collective catering, or, alternatively, a “single course” can be proposed, including both foods rich in carbohydrates (typically consisting of cereals) and foods with a good protein composition (legumes). First courses intended for collective catering (school canteens and healthcare facilities) must be made up of cereals (pasta, rice, barley, corn, etc.) and prepared using a variety of different recipes respecting local traditions and replacing dry first courses and soups. As an alternative to pasta and rice, first courses based on barley, spelt, oats, cornmeal and quinoa are suggested, often associated with greens, vegetables and legumes, useful to allow a wide variety of flavors and a reduction in the glycemic index. A valid replacement for dry first courses is vegetable soup, to be served at least once a week, especially in winter. Furthermore, it is useful to frequently promote the use of wholegrains rich in fiber and micronutrients.

The second course should consist of white meat (chicken, turkey and rabbit), red meat (veal, beef and pork), fish, meat-based products (e.g., cooked, raw ham or bresaola), eggs and cheeses, with suitable preparations for all age groups, promoting an adequate rotation. Due to the fatty acid content, following the indications provided by the Italian guidelines for a healthy diet, cured meats should be limited and possibly proposed as a replacement for red meat.

In general, each meal should include a side dish of vegetables of at least three different types during the week, alternating between cooked and raw; bread with no added fat and with a reduced salt content; and seasonal fruit of at least three different types during the week, if ready to eat. Potatoes are not considered vegetables and should be offered as a first course once every two weeks. For the dressing, for both raw and cooked food, only extra virgin olive oil should be added, due to its recognized nutraceutical value. Aromatic herbs should also be used, while limiting butter and salt intake. Moreover, iodized salt is preferred to other salts. The use of bouillon cubes containing monosodium glutamate or any other food matrix containing it is prohibited.

The general aim of this study is to evaluate the conformity of school and healthcare facility menus to the new regional guidelines for schools, companies and hospital caterers. The study was carried out in a five year period between 2018 and 2022 by the Food Hygiene and Nutrition Service (SIAN) of the Local Health Authority ASL BT (LHA), as the competent body for official controls. The specific aims of the investigation are targeted to: (i) assess the compliance of the menus adopted in both school catering services and healthcare facilities, subject to endorsement by the SIAN staff of LHA; (ii) analyze the types of non-compliance detected, hypothesizing the origin and suggesting corrective actions; and (iii) evaluate the need for subsequent on-site official controls in order to evaluate the conformity between the endorsed menu and the type of meals actually administered.

## 2. Materials and Methods

Overall, in the BT province, 72 comprehensive institutes (CIs) and didactic circles (DCs) benefit from a school canteen service. The 72 CIs and DCs are school aggregation units, including 218 schools of any type or level. In the same territorial context, 18 accredited healthcare facilities are present, which are hospitals or private structures that provide services on behalf of the National Health Service.

**Figure 1:**
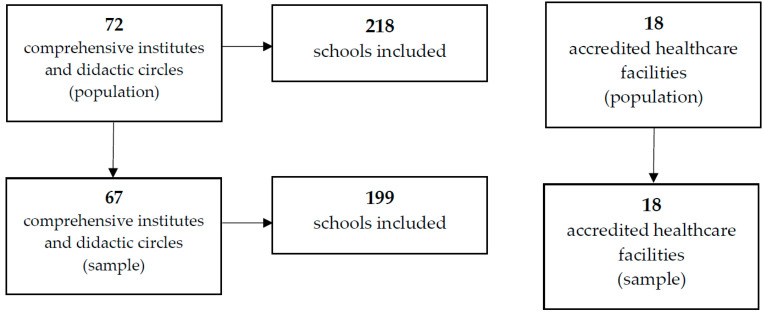
Graphical representation of the enrollment method for both schools and healthcare facilities.

In the years 2018–2022, the SIAN of ASL Barletta–Andria–Trani (BT) LHA received 85 requests for menu endorsements, of which 67 were from CIs/DCs that contained a total of 199 school units and 18 were from accredited healthcare facilities. Hence, as represented in Figure 1, the sample investigated is representative of 93.05% of the CIs/DCs (91.3% of the school units) located in the reference territory and of the entirety (100%) of accredited healthcare facilities.

All menus, edited by the professionals of each structure (e.g., nutritionists and dieticians), were submitted to the LHA for assessment by SIAN staff. Checklists with the requirements to be met were used in order to precisely evaluate the menus proposed.

In compliance with the regional guidelines for school catering, menus must be specifically edited to meet the needs of pre-school and school-age subjects [16,18]. With regard to healthcare facilities, menu contents were compared with the national guidelines for hospital and healthcare catering based on two cornerstones: the Mediterranean lifestyle and the fulfillment of the nutritional caloric intake in order to prevent malnutrition. Furthermore, the regional guidelines for hospital catering were also considered since, unlike the national ones, they provide a greater level of detail for the type of food to be offered and for the frequency at which they should be offered, inspired by the Mediterranean model adhered to by the reference target population [17,19].

All menus must be divided into two seasonal versions, one in autumn–winter, for the months from October to March, and one in spring–summer, for the period from April to September, structured taking into account a differentiation over four weeks (one month).

The proposed menus were evaluated and the results were reported in a database using Microsoft Excel©. All statistical data analyses were carried out using the software R version 4.0.3 (10 October 2020) (“Bunny Wunnies Freak Out” Copyright© 2020 The R Foundation for Statistical Computing). The normality of all the variables was evaluated with a D’Agostino–Pearson normality test. Categorical data were represented as numbers (n) and percentages (%). The χ2 test was applied to evaluate the difference in proportions. The level of significance, indicating evidence against the null hypothesis for all statistical tests performed, was set at *p* < 0.05.

## 3. Results

The processing of results relating to the menus specifically for use in school catering shows numerous critical issues in relation to the compliance observed, as shown in Table 1, where the differences statistically significant are shown in bold.

All schools included legumes on the menu, and no school decided to benefit from the choice of using cereal grains or wholegrain pasta and bread. Only 8.9% of schools did not use prepacked foods such as fish fingers, just 11.9% of schools respected the correct food weight and only 17.9% rotated seasonal fruit and vegetables.

Overall, 34.3% have cooked ham on the menu, in more than half of the schools a single course is provided and in 88% of the schools potatoes are correctly considered a substitute for pasta and not a side dish. Almost 90% of schools have menus divided into autumn–winter and spring–summer seasons.

The results of the evaluation of the menus applicable to healthcare facilities are listed in Table 2, where the differences statistically significant are shown in bold.

All the institutions investigated offered legumes on their menus. Only two food establishments out of 18 provided both grain cereals and a single course on the menu, just three establishments provided wholegrain pasta and bread and only one-third of establishments did not use prepacked foods.

Almost half of the structures had cooked ham and potatoes on the menu as a first course, only partially respecting the seasonality of fruit and vegetables. Overall, 77.7% of healthcare facilities respected the seasonal subdivision of the menus into autumn–winter and spring–summer and more than 80% of the establishments served the correct food weight.

Analyzing the data derived from school catering and healthcare facilities, wide divergences emerge between the expected data and the observed data. The statistical tests also show significant differences between the expected data in accordance with the guidelines and the data observed and assessed by the SIAN staff.

## 4. Discussion

### 4.1. Outcomes of the Investigation

A correct and balanced diet is an essential requirement for people’s well-being. From the data collected and examined in our study for school catering, the intake of legumes is fully compliant with the consumption recommended by the regional guidelines. The additional foods investigated and evaluated show statistically significant non-compliances. Potatoes, a source of complex carbohydrates, continue to be included in menus as a side dish, even though they are not vegetable and, consequently, they should be offered as a replacement for pasta and other cereals, both as a first course or in association with a meal lacking in additional carbohydrates. Cereal grains such as barley, spelt, quinoa or wholemeal bread and pasta, usually belonging to the Mediterranean food model and essential for their fiber and micronutrient (vitamins and mineral salts) contents, were not included in any presented menu. A single course, sufficient to cover the energy and nutritional needs of a complete meal (e.g., pasta and beans), is only partially available in the examined menus, despite the guidelines recommending its constant presence at least once per week. The presence of prepacked products further indicates the failure to adhere to the guidelines, which recommend the exclusion of processed and packaged products. Among these, fish fingers and fish sticks are commonly used above all in school canteens. These foods consist of fish-based products with a high percentage of carbohydrates, saturated fats and trans fatty acids compared to the contents of fresh fish, as they are ultra-processed products, breaded with breadcrumbs, wheat flour or potato starch and herbs, then fried with vegetable oils, which are often not even specifically indicated among the ingredients. The high palatability of these foods is not only as a result of the oil used for frying, but also a result of the high salt content. Therefore, it is a pre-fried food with a higher content of carbohydrates than proteins, it is rich in fat even when cooked in the oven and it is highly caloric compared to a fresh or frozen fish fillet; moreover, it is high in salt and low in omega-3 polyunsaturated fatty acids. In addition, the weekly amount of cooked ham served on menus is not compliant, resulting as too high. Considering the high amount of sodium, saturated fats, cholesterol and additives, such as nitrites and nitrates, monosodium glutamate and polyphosphates, this food matrix should be offered no more than twice a month.

Furthermore, the incorrect subdivision between autumn–winter and spring–summer menus consequentially means that the seasonality of fruit and vegetables is not fully respected too.

The seasonality of the menus is not adequately respected, although particular attention is recommended to ensure the seasonal rotation of vegetables when choosing vegetable side courses. This topic is currently regulated in our country by the official document *Criteri Ambientali Minimi* (CAM)—Minimum Environmental Criteria for the collective catering service and the supply of foodstuffs [20].

Recent studies have investigated the nutritional quality of menus adopted in schools located in other countries, highlighting non-compliances attributable to a lack of healthier options and highlighting a lack of macro- and micro-nutrients and a high salt content. By promoting specific projects and carrying out official follow-up controls, in some virtuous contexts it has been proven that compliance with respect to the guidelines appears to be very high [21,22,23]. In our study, the non-compliance detected could also be attributable to the supply management procedure. Indeed, the FBO should choose its suppliers based on specific criteria and qualifications, drawing up management procedures to monitor compliance with and maintenance of the selection criteria set out in the purchase specifications.

Notably, one further non-compliant criterion observed concerns the food weight, which did not always comply with the guidelines. Respecting weight is of fundamental importance to guarantee that children have the right caloric intake, which is connected to height and weight growth according to different age groups. Providing an adequate food quantity is essential for receiving the correct nutrition and achieving the right energy requirements if placed in a context based on prevention and health promotion. This approach is undoubtedly useful for school-age children benefiting from a canteen service in order to promote a sense of awareness with respect to the appropriate quantities of food to be eaten daily. However, administering food in adequate quantities can be difficult, so it is essential that the canteen personnel are fully aware of the reference portions. In order to make this activity easy and truly implementable, the portion, as well as the weight, must be calculated by nutrition professionals and adapted to the needs of each individual, taking into account gender, age, physical activity and health status. Resorting to the aid of a scale is not always possible in operational terms; consequently, the need to train food business operators (FBOs) arises. It would also be advisable to create operating instructions with photo guides, useful to quickly highlight the compliance between the action performed by FBOs during the plating phase and the document stating the reference quantities. The regional guidelines indicate the ideal weights of each food, enclosed in a summary table that makes it possible to identify in an intelligible way the correct amount of food expected for subjects at each development stage.

Similar to school catering, the same non-conformities emerged in healthcare facilities, with the exception of legumes and weights. Sure enough, these last items assessed met the specifically recommended energy and nutrient needs [24,25].

It should be noted that school catering cannot be considered as the mere satisfaction of nutritional needs, but as a moment of education and health promotion. Eating at school represents a convivial moment, useful for enriching the home food model through new flavors, tastes and food experiences and managing the difficulties some children have with food they have never eaten—which are often protective in terms of health—or food with a taste not appreciated for the first time. Meals served in the school canteen offer greater guarantees both in terms of hygiene and nutrition and are preferred over the consumption of food brought from home as they are more susceptible to targeted official controls [26].

If for the school-age population the acquired healthy food style could be maintained for life, the correct nutrition in healthcare facilities is even more essential, since it correlates with a favorable prognosis for the patient. Malnutrition is common in hospitalized/institutionalized patients, especially in frail and elderly people with cognitive impairment, and negatively affects prognosis. In this population, it has been demonstrated that malnutrition worsens the quality of life and increases morbidity and mortality [27]. It has been extensively demonstrated that a balanced nutrition can affect the disease progress of the sick person, also resulting in a cost saving for health care homes [28,29].

### 4.2. Practical Implications and Sustainability in Food Businesses

From our investigation, it is evident that in order to adequately respond to the provisions of the regional guidelines, FBOs should implement multiple activities aimed at continuous improvement, taking into account various aspects in order to ensure compliance with the sector legislation. In detail, FBOs assigned to food delivery must carry out direct actions on supplies through:

- Visual checks of the hygienic sanitary state of the raw material;
- Visual checks of label information (e.g., an indication of the expiry date, allergens, etc.);
- An assessment of compliance with the specifications indicated on the transport document or invoice (product name, quantities sold and correspondence of the lot reported);
- The control of transport temperatures for perishable products;
- Congruity with respect to the type of product ordered (e.g., seasonal vegetables replaced with something else);
- Internal laboratory checks [30,31,32,33].

Menu verification should also take into consideration some matters, in particular the perceived quality through the periodic collection of information relating to consumer satisfaction, which can be used as a basis for inducing improvements; the evaluation of surpluses and residues through a quantitative and qualitative assessment of food not distributed, both single-portion and multi-portion; and the courses rejected by consumers, for the redefinition of weights, portions, recipes and sensory characteristics.

For catering businesses, it would also be advisable to adopt an information technology (IT) management tool, useful to guide and monitor all production stages, starting from goods delivery to creating a virtual warehouse suitable for providing information on the lot, expiry dates and products in stock, facilitating the rotation of raw materials at the same time. Depending on the daily menu, the application could have multiple purposes, e.g., scheduling the supply; indicating the weight of each course; suggesting previously endorsed menus according to the seasonality; implementing blockchain mechanisms for goods management and control; and daily tracing of raw materials and the destination of the final product up to it being served.

A list of qualified suppliers, including data useful for assessing their reliability and qualification criteria, must be created, taking into account the history of the collaboration; test orders; certifications related to the quality and safety of the food product; and the results of audits directly conducted at the supplier’s establishments. For the purpose of a correct supply in compliance with the rotation and seasonality of the products, it is essential to monitor order fulfillment times, which is useful for guaranteeing the on-time arrival of goods.

Sustainable diets have a low environmental impact and contribute to food and nutritional safety, as well as to a healthy life for present and future generations. Moreover, this approach contributes to both the respect of biodiversity and ecosystems, optimizing natural and human resources [34]. In addition to generating a significant economic and social impact, food waste puts undue pressure on limited natural resources and on the environment. Approximately one-third of the world’s annual food production intended for human consumption ends up being lost or wasted in some way [35]. In the presence of surpluses, redistribution to the human food supply is the best possible destination, guaranteeing the highest use value of food resources suitable for consumption.

For the prevention and management of food surpluses, it is useful to carry out calculations and continuous monitoring. Subsequently, the reasons underlying why any excess is generated should be analyzed by means of questionnaires, for example (e.g., satisfaction questionnaires to detect user satisfaction level). The reduction in food waste is possible through the implementation of various strategies, such as the correct structuring of menus, the improvement of organoleptic qualities and the preparation of targeted foods [36,37,38].

In order to increasingly reduce food waste, the European Commission has recently proposed to integrate and amend the Directive 2008/98/EC. In particular, it has been suggested to add the wording “often good after” next to “best before”. This will allow eventual benefits from this feature during the supply phase. In any case, this would not exempt the FBO from evaluating any risks to which the product may be exposed, from the packaging integrity to any other aspect that may affect the condition of food, which must be suitable for human consumption [39]. Taking into account the environmental and cost impact of a canteen meal and food waste management at schools represents a significant future challenge [40]. Recent sector studies performed in our region have already documented the existence of specific projects aimed at promoting food education intervention on this topic [41]. FBOs can join different initiatives that donate food surplus to organizations that can get good quality surplus onto the plates of vulnerable people.

### 4.3. Study Limits and Future Perspectives

Although our investigation is representative of the collective catering activities at the local level, it deals with a provincial area of reference. Therefore, the results cannot be extended either to the regional or national level, where, in addition, the norms can be different. Furthermore, at present, our study is based on the evaluation of menus for LHA documental endorsement in a preventive phase. Follow-up studies are necessary to assess the effective implementation of the suggested correction measures over time and to evaluate on site the correspondence between the foods on the daily menus and the foods actually served.

Nevertheless, to the best of our knowledge, this is the first study that evaluates the compliance of the proposed menus with specific regional guidelines. It is essential to highlight that, at this preliminary level of checks, only a partial compliance with the provisions of the law has emerged. It is therefore essential to plan and continue promoting actions in this field, aimed at verifying the activities undertaken by these types of food businesses. It should be noted that professionals in this field always drew up the analyzed menus; nevertheless, without official controls performed by the LHA, they would have reached the tables of collective catering users with significant critical outcomes. This underlines the importance of both controls and the monitoring performed by SIAN on one hand, and, on the other, the need for dedicated training events to make freelance professionals aware of the correct way to draft menus. From these findings, it follows that there is a need to continue to strengthen the role of the Services of Prevention Departments dedicated to food and nutrition safety, to continue to invest in terms of employing professionals with a multidisciplinary background and to continue targeted training to guarantee joint official controls performed by physicians, biologists, dieticians, food technologists, health visitors and environmental health officers [42,43].

## 5. Conclusions

In school canteens and in healthcare facilities, providing and administering food is an opportunity to promote health through a balanced diet and safe food, offering the prospect of the development of the community’s well-being and local economy in a sustainable way. The collective catering sector is of particular importance, which is directing increasing attention to the issues of the quality and sustainability of products offered to consumers according to the principles of “catering promoting health”. Our study reveals critical issues regarding the drafting of menus that are not always in compliance with the guidelines. Therefore, it is essential for the LHA to evaluate the proposed menus, followed by official controls on both the compliance of the menus during the daily food delivery and on the hygienic conditions of schools and healthcare facilities [26,44]. At the same time, it is important for policy makers to provide harmonized guide documents, taking into account the legislation and also the peculiarities of local food production.

Finally, both nutrition professionals responsible for drafting menus and FBOs must be adequately trained, to meet the minimum requirements for collective food services and projects to manage food surpluses.

## Figures and Tables

**Table 1:** Evaluation of menus intended for school catering.

Foods or Items Assessed	Observed Compliance	Expected Compliance	*p*-Value
legumes, n (%)	67 (100)	67 (100)	-
potatoes, n (%)	59 (88.0)	67 (100)	**0.003**
prepacked food (fish fingers), n (%)	6 (8.9)	0 (0)	**0.01**
single course, n (%)	38 (56.7)	67 (100)	**<0.0001**
cereal grains, n (%)	0 (0.0)	67 (100)	**<0.0001**
wholegrain pasta and bread, n (%)	0(0.0)	67 (100)	**<0.0001**
ham steak, n (%)	23 (34.3)	67 (100)	**<0.0001**
fruit and vegetables rotation, n (%)	12 (17.9)	67 (100)	**<0.0001**
weight, n (%)	8 (11.9)	67 (100)	**<0.0001**
seasonality, n (%)	60 (89.5)	67 (100)	**0.006**

**Table 2:** Evaluation of menus intended for healthcare facilities.

Food or Items Assessed	Observed Compliance	Expected Compliance	*p*-Value
legumes, n (%)	18 (100)	18 (100)	-
potatoes, n (%)	8 (44.4)	18 (100)	**0.002**
prepacked food (fish fingers), n (%)	6 (33.3)	0 (0)	**0.0073**
single course, n (%)	2 (11.1)	18 (100)	**<0.0001**
cereal grains, n (%)	2 (11.1)	18 (100)	**<0.0001**
wholegrain pasta and bread, n (%)	3 (16.6)	18 (100)	**<0.0001**
ham steak, n (%)	8 (44.4)	18 (100)	**0.002**
fruit and vegetables rotation, n (%)	8 (44.4)	18 (100)	**0.002**
weight, n (%)	15 (83.3)	18 (100)	0.070
seasonality, n (%)	14 (77.7)	18 (100)	**0.033**
